# CRISPR/Cas12a toolbox for genome editing in *Methanosarcina acetivorans*

**DOI:** 10.3389/fmicb.2023.1235616

**Published:** 2023-12-12

**Authors:** Ping Zhu, Tejas Somvanshi, Jichen Bao, Silvan Scheller

**Affiliations:** Laboratory of Biochemistry, Department of Bioproducts and Biosystems, School of Chemical Engineering, Aalto University, Espoo, Finland

**Keywords:** CRISPR/Cas12a, genome editing, *Methanosarcina acetivorans*, methanogens, synthetic biology

## Abstract

Methanogenic archaea play an important role in the global carbon cycle and may serve as host organisms for the biotechnological production of fuels and chemicals from CO_2_ and other one-carbon substrates. *Methanosarcina acetivorans* is extensively studied as a model methanogen due to its large genome, versatile substrate range, and available genetic tools. Genome editing in *M. acetivorans* via CRISPR/Cas9 has also been demonstrated. Here, we describe a user-friendly CRISPR/Cas12a toolbox that recognizes T-rich (5′-TTTV) PAM sequences. The toolbox can manage deletions of 3,500 bp (i.e., knocking out the entire *frhADGB* operon) and heterologous gene insertions with positive rates of over 80%. Cas12a-mediated multiplex genome editing was used to edit two separate sites on the chromosome in one round of editing. Double deletions of 100 bp were achieved, with 8/8 of transformants being edited correctly. Simultaneous deletion of 100 bp at one site and replacement of 100 bp with the 2,400 bp *uidA* expression cassette at a separate site yielded 5/6 correctly edited transformants. Our CRISPR/Cas12a toolbox enables reliable genome editing, and it can be used in parallel with the previously reported Cas9-based system for the genetic engineering of the *Methanosarcina* species.

## Introduction

Methanogenic archaea are significant global contributors to the production of the potent greenhouse gas methane, which is estimated to be about 1 Gt/year (Thauer et al., 2010). At the same time, these microorganisms are increasingly utilized for the biotechnological reduction of atmospheric CO_2_, and therefore may help mitigate global warming. *Methanosarcina* species are among the best studied, as many details about their genomic information (Deppenmeier et al., 2002; Galagan et al., 2002; Maeder et al., 2006) and versatile methanogenesis (Costa and Leigh, 2014; Kurth et al., 2020) have been earlier revealed. Metabolic engineering of *Methanosarcina* facilitates many diverse biotechnological applications. For instance, the engineered expression of an esterase in *Methanosarcina acetivorans* allowed cells to utilize methyl esters for growth and methane production (Lessner et al., 2010). Additionally, isoprene production was demonstrated by engineering the expression of an isoprene synthase gene in *Methanosarcina* (Aldridge et al., 2021).

Genome manipulations in *M. acetivorans* are typically more challenging than those in well-established host microorganisms (e.g., *Escherichia coli*) because of the multiple copies of its genome (Hildenbrand et al., 2011). If not all genome copies are uniformly edited, heterozygous mutants that still retain a wild-type chromosome may persist. For metabolic engineering projects requiring several rounds of editing, marker-free edits are necessary. In *Methanosarcina*, the puromycin acetyltransferase (*pac*) gene for puromycin resistance and the isoleucyl-tRNA synthetase (*ileS*) gene for pseudomonic acid resistance are the two selective markers currently used (Metcalf et al., 1997; Boccazzi et al., 2000). The hypoxanthine phosphoribosyltransferase (*hpt*) gene has been widely utilized as a counterselectable marker in *Methanosarcina* Δhpt hosts (Guss et al., 2008). Traditional gene editing, which involves introducing linear DNA fragments followed by homologous recombination, generally shows lower transformation and editing efficiencies than CRISPR (clustered regularly interspaced short palindromic repeats)-based methods (Ronda et al., 2016). The ΦC31 integrase-mediated site-specific recombination system, which has a fixed target site on the genome (Guss et al., 2008), is efficient for introducing a singular piece of DNA, but lacks the ability to edit multiple sites or perform repeated edits. As an alternative, CRISPR system-mediated genome editing tools have been developed for methanogens. The CRISPR/Cas9 system was initially generated for *M. acetivorans* (Nayak and Metcalf, 2017), followed by the construction of the CRISPRi-dCas9 tool for gene regulation (Dhamad and Lessner, 2020) in the same methanogen.

Cas12a (also known as Cpf1) is a class 2 type V endonuclease (Paul and Montoya, 2020) that recognizes a thymine (T)-rich (5′-TTTV, V = A, G, and C) protospacer adjacent motif (PAM) (Safari et al., 2019), as opposed to the guanine (G)-rich (NGG-3′) PAM of Cas9. Consequently, Cas12a increases the number of potential targeting sites along the genome, especially for T-rich hosts. The double-stranded breaks (DSB) caused by Cas12a will generate sticky ends, which are helpful for DNA repair and genome stability during genetic manipulation (Vanegas et al., 2019). Since the gRNA (guide RNA, also called spacer) does not rely on tracrRNA (trans-activating CRISPR RNA) for maturation (Nakade et al., 2017), edited constructions become simplified, particularly when multiplex genome editing is employed (Bandyopadhyay et al., 2020). More recently, a Cas12a-based genome editing system has been successfully implemented for genetic engineering in *Methanococcus maripaludis* (Bao et al., 2022).

To increase the versatility of CRISPR editing in *M. acetivorans*, we constructed a system that expresses the Cas12a endonuclease from *Lachnospiraceae bacterium* (LbCas12a). To evaluate the performance of this editing toolbox, single gRNA-mediated gene knockout and heterologous gene integration approaches were used. As a tool to expedite metabolic engineering, Cas12a-mediated multiplex genome editing was to establish gene edits at two independent sites along the genome with only one round of editing.

## Results

### Construction of Cas12a-gRNA expression system

**Figure 1:**
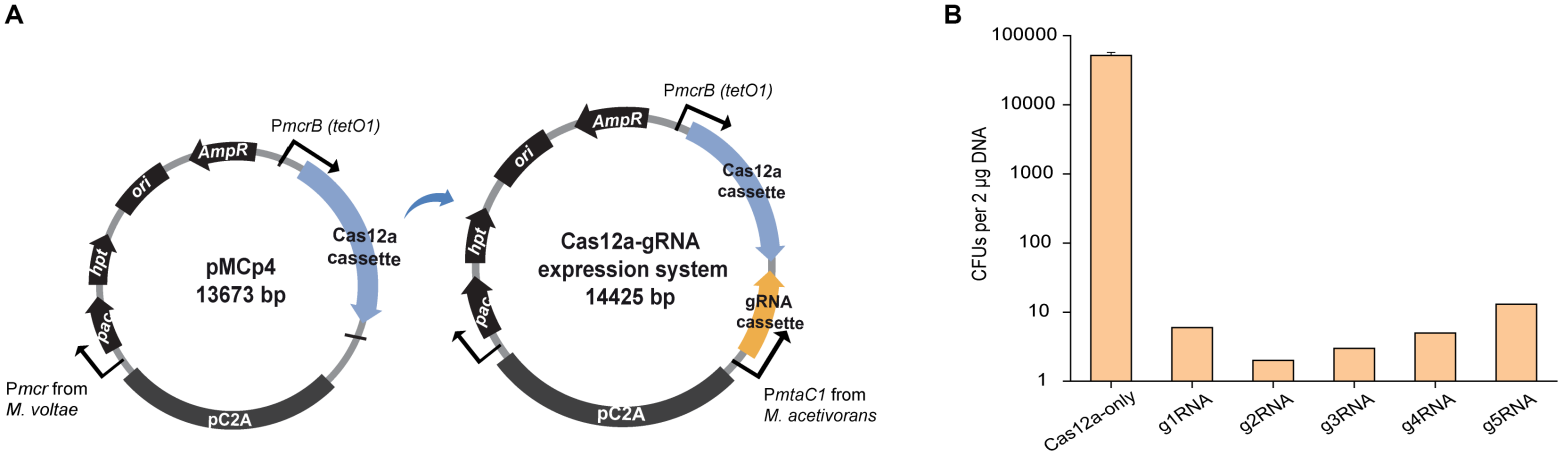
Construction of the Cas12a-gRNA expression system for *M. acetivorans*. **(A)** Schematic diagram of pMCp4 and Cas12a-gRNA expression system. The plasmid pMCp4 expresses the Cas12a protein in *M. acetivorans*. The Cas12a-gRNA expression system expresses the Cas12a-gRNA complex in *M. acetivorans*. Cas12a and gRNA cassettes are equipped with tetracycline-regulated promoter P*mcrB* (*tetO1*) and the promoter P*mtaC1* from *M. acetivorans*, respectively. **(B)** Targeting efficiency of the Cas12a-gRNA expression system. Cas12a-only, plasmid pMCp4. g1RNA, g2RNA, g3RNA, g4RNA, and g5RNA, five gRNAs designed for targeting various locations along the *ssuC* gene. Error bar represents the standard deviation of triplicate measurements. Standard deviations were not determined for gRNA-expressing transformation data, as all cells were plated out to analyze the lethal efficiency of the Cas12a-gRNA complex.

To generate a Cas12a-gRNA expression system that is cloneable in most *E. coli* strains, the *E. coli/Methanosarcina* shuttle vector pM000 (Supplementary Figure S1B) was constructed from the pWM321 plasmid (Metcalf et al., 1997) by replacing the origin of replication (*ori*) from plasmid R6K with the *ori* from plasmid ColE1. To establish markerless editability, the *hpt* gene that confers sensitivity to the purine analog 8-aza-2,6-diaminopurine (8ADP) for counter selection (Ehlers et al., 2011) was inserted downstream of the *pac* gene, yielding plasmid pM001 (Supplementary Figure S1C). The *LbCas12a* gene was amplified from pY016 (Zetsche et al., 2015), fused with the tetracycline-regulated promoter P*mcrB* (*tetO1*), and inserted into the multiple cloning site (MCS) of pM001 to yield the plasmid pMCp4. To obtain the final Cas12a-gRNA expression system, the gRNA cassette is introduced into pMCp4 (Figure 1A). It is worth mentioning that tetracycline was not required for the expression of Cas12a, as in the previous studies with Cas9, where no significant difference in the genome editing efficiency was observed in *M. acetivorans* (Nayak and Metcalf, 2017; Dhamad and Lessner, 2020).

To verify whether the Cas12a protein is not cytotoxic, the shuttle vector pM001 and the Cas12a-expressing plasmid pMCp4 were each transformed into *M. acetivorans*, and then the transformation efficiencies were compared. When 2 μg of plasmid DNA was used for the transformations, pM001 yielded 40,000 ± 20,000 CFUs of Pur^R^ transformants and pMCp4 yielded 34,000 ± 19,000 CFUs of Pur^R^ transformants (*p* = 0.75 in two-tailed *t*-test, Supplementary Figure S2), which indicates the expression of Cas12a protein is non-toxic to *M. acetivorans* cells.

To evaluate the targeting efficiency of the Cas12a-gRNA expression system, five different gRNAs were used to target various locations along the non-essential *ssuC* gene (which encodes the permease subunit of the sulfonate ABC transporter) for introducing DSBs on the genome. All five gRNAs resulted in less than 15 CFUs (per 2 μg DNA) in the transformation, thus demonstrating the high gene-targeting efficiency of the constructed system (Figure 1B).

### CRISPR/Cas12a-mediated gene knockout

**Figure 2:**
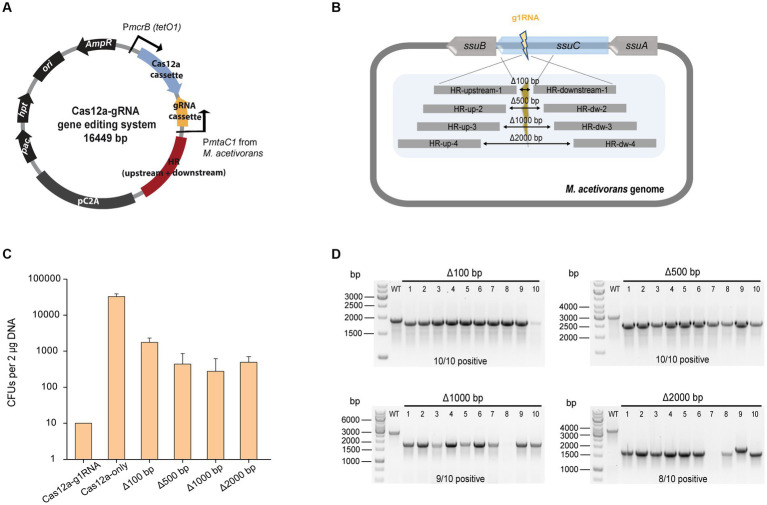
CRISPR/Cas12a-mediated gene knockout. **(A)** Schematic diagram of Cas12a-gRNA gene editing system. Cas12a and gRNA cassettes are equipped with promoters P*mcrB* (*tetO1*) and P*mtaC1*, respectively. Gene editing was achieved by the introduced upstream and downstream homologous repair (HR) arms. **(B)** Scheme for generating gene deletions in *ssuC*. g1RNA was designed to target *ssuC* to form a double-stranded break (DSB). Various sizes of gene knockouts were generated by introducing a 1,000-bp length of flanking HR sequence near the leakage site. Δ100 bp, Δ500 bp, Δ1000 bp, and Δ2000 bp, plasmids generating 100 bp, 500 bp, 1,000 bp, and 2000 bp of gene knockouts while repairing the DSB. **(C)** Transformation efficiency of deletion-generating plasmids. Cas12a-g1RNA, plasmid expressing Cas12a-g1RNA complex that targets *ssuC* to produce the DSB on the genome. Cas12a-only, plasmid pMCp4 expresses Cas12a. Error bars represent the standard deviation of triplicate measurements. Standard deviations were not determined for Cas12a-g1RNA transformation data, as all cells were plated out to analyze the lethal efficiency of the Cas12a-g1RNA complex. **(D)** Editing efficiency of deletion-generating plasmids. Ten Pur^R^ transformants were randomly selected for colony PCR. WT, wild type *M. acetivorans* strain. Thermo Scientific™ GeneRuler 1 kb DNA ladder was used for sizing DNA fragments.

To investigate the efficiency of gene deletion, the homologous repair (HR) arms for gene editing were inserted into the Cas12a-gRNA expression system to generate the Cas12a-gRNA gene editing system (Figure 2A). The g1RNA (5’-TTGCGATTCCCTCAGCCATG CCC-3′) was used to target the *ssuC* locus. For gRNA cassette construction, PCR amplification was used to synthesize the gRNA and direct repeat (DR) sequences that contain promoter (P*mtaC1*) and terminator (T*mtaB1*) regions (Supplementary Figure S3A). Deletion lengths varied from 100 bp to 2000 bp (Figure 2B) and a 1,000 bp length of flanking HR sequence was chosen as optimal based on previous work (Nayak and Metcalf, 2017).

When the plasmid pMCp2-g1RNA (Table 1) was transformed into *M. acetivorans*, the Cas12a-g1RNA complex was expressed and only 10 Pur^R^ transformants were observed (Figure 2C). Transformation of plasmid pMCp4 (Cas12a-only) yielded 33,000 ± 6,000 CFUs of Pur^R^ transformants (per 2 μg DNA). Among the various lengths of deletions tested, the 100 bp-deletion from the genome gave the highest repairing efficiency (1,800 ± 600 CFUs). This higher transformation efficiency obtained when only a small fragment of the chromosome is deleted also matches a previous Cas9-mediated gene knockout study, which concluded shorter deletions (i.e., Δ100 bp and Δ500 bp) are more stable and reliable (Nayak and Metcalf, 2017). For the plasmids that led to larger fragment deletions (i.e., Δ1000 bp and Δ2000 bp), similar transformant yields were obtained. To verify the knockout (or editing) efficiency, 10 transformants were randomly selected in each trial and subjected to colony PCR (Figure 2D). The primers involved are presented in Supplementary Figure S4A and Supplementary Table S1. Higher positive rates were observed with the shorter deletions, in which 100% of the 100 bp-deleted transformants were positive. On the other hand, 80% of positive clones were observed in the 2000 bp-deletion experiments. Three isolates from each transformation set were randomly selected for Sanger sequencing and all were edited correctly (Supplementary Figure S4B). The Sanger sequencing results were provided in Supplementary Table S2.

**Table 1:** Plasmids used in this study.

Plasmid	Description	Source
pY016	gene sequence source of LbCas12a	Zetsche et al. (2015)
pWM321	*Escherichia coli*/*Methanosarcina* shuttle vector	Metcalf et al. (1997)
pM000	pWM321-derived vector, ColE1 origin of replication (high-copy-number from *E. coli*)	This study^1^
pM001	pM000-derived vector with hypoxanthine phosphoribosyltransferase (*hpt*) gene	This study^2^
pMCp4	pM001-derived plasmid with Cas12a cassette	This study^3^
pMCp2-g1RNA	pMCp4-derived plasmid with g1RNA cassette, targeting *ssuC* locus	This study^4^
pMCp3-g1-100	pMCp2-g1RNA-derived plasmid with flanked homologous repair arms generating 100 bp of deletion on genome	This study^5^
pMCp3-g1-500	pMCp2-g1RNA-derived plasmid with flanked homologous repair arms generating 500 bp of deletion on genome	This study^6^
pMCp3-g1-1000	pMCp2-g1RNA-derived plasmid with flanked homologous repair arms generating 1,000 bp of deletion on genome	This study^7^
pMCp3-g1-2000	pMCp2-g1RNA-derived plasmid with flanked homologous repair arms generating 2000 bp of deletion on genome	This study^8^
pMCp2-g9RNA	pMCp4-derived plasmid with g9RNA cassette, targeting *frhA* locus	This study^9^
pMCp2-gX	pMCp2-g1RNA-derived plasmid with *AarI* digestion sites removed, g1RNA sequence replaced with two *AarI* sites and one *NotI* site	This study^10^
pMCp3-g9-3500	pMCp2-g9RNA-derived plasmid with flanked homologous repair arms generating 3,500 bp of deletion on genome	This study^11^
pMCp3-g1-100-uid	pMCp2-g1RNA-derived plasmid with flanked homologous repair arms with *uidA* expression cassette inserted in genome	This study^12^
pMCp2-g1g9RNA	pMCp4-derived plasmid with g1g9RNA expression cassette, targeting *ssuC* and *frhA* genes simultaneously	This study^13^
pMCp3-g1g9–100	pMCp2-g1g9RNA-derived plasmid with two sets of homologous repair arms generating 100 bp deletions on *ssuC* and *frhA* locus simultaneously	This study^14^
pMCp3-g1-uid-g9-100	pMCp2-g1g9RNA-derived plasmid with two sets of homologous repair arms with *uidA* expression cassette inserted in *ssuC* locus and 100 bp deletion in *frhA* locus simultaneously	This study^15^

The plasmid sequences are available via links in Supplementary Presentation 1.

### CRISPR/Cas12a-mediated gene insertion

**Figure 3:**
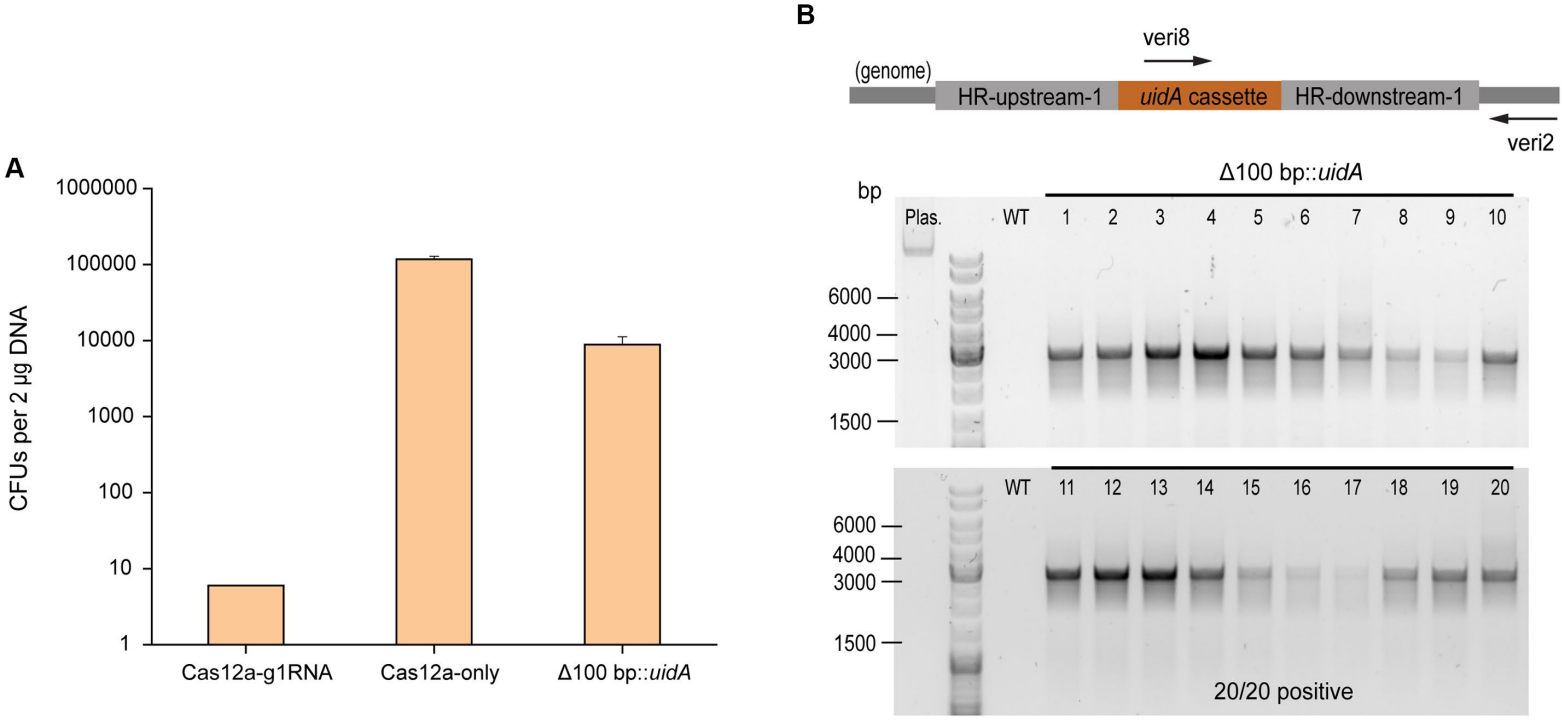
CRISPR/Cas12a-mediated gene insertion. **(A)** Transformation efficiency of Cas12a-mediated gene insertion. Cas12a-g1RNA, plasmid pMCp2-g1RNA expressing Cas12a-g1RNA complex that targets *ssuC* to produce the DSB on the genome. Cas12a-only, plasmid pMCp4 expresses Cas12a. Δ100 bp::*uidA*, plasmid pMCp3-g1-100-uid replaced 100 bp with the *uidA* expressing cassette in genome. Error bars represent the standard deviation of triplicate measurements. Standard deviations were not determined for Cas12a-g1RNA transformation data, as all cells were plated out to analyze the lethal efficiency of the Cas12a-g1RNA complex. **(B)** Editing efficiency of gene insertion-generating plasmid. Upper panel, scheme for the engineered genome containing *uidA* cassette and the detecting primers used in colony PCR. veri8 and veri2, forward and reverse primer target *uidA* and genome. HR-upstream-1 and HR-downstream-1, the flanking HR sequence identical to the ones used in Figure 2B. Bottom panel, 20 Pur^R^ transformants were randomly selected for colony PCR to verify the existance of the *uidA* gene. Plas. and WT are plasmid pMCp3-g1-100-uid and wild type *M. acetivorans* genome, respectively, and served as negative controls. Thermo Scientific™ GeneRuler DNA Ladder Mix was used for sizing DNA fragments.

In order to assess the gene insertion performance of our Cas12a-gRNA gene editing system, a heterologous gene cassette for the expression of β-glucuronidase (*uidA*) gene was placed within the HR arms, given its previous successful use in *Methanosarcina* (Guss et al., 2008). For *uidA* cassette construction, the *uidA* gene was PCR amplified from *E. coli* BL21 genomic DNA and fused with the *mcr* promoter (P*mcr*) and terminator (T*mcr*) from *M. barkeri* (Supplementary Figure S5A). The insertion efficiency was obtained by comparing the number of Pur^R^ transformants from the editing plasmid pMCp3-g1-100-uid (8,800 ± 2,400 CFUs) and plasmid pMCp4 (117,000 ± 12,000 CFUs) (Figure 3A). The positive rate for 20 transformants was determined by colony PCR using the primers veri2/veri8 (Figure 3B). To ensure the genome copies within one transformant were all edited (homozygous chromosome), a second set of primers (veri5/veri12) was used to detect the presence of possible mixed transformants (Supplementary Figure S5B). 100% of the transformants were identified with *uidA* cassette and no heterozygous genotypes were detected. Three isolates were randomly selected for Sanger sequencing to detect the *uidA* gene cassette and all the edits were positive (Supplementary Figure S5C and Supplementary Table S2), demonstrating the high performance for Cas12a-mediated gene insertion.

### CRISPR/Cas12a-mediated multiplex genome editing

**Figure 4:**
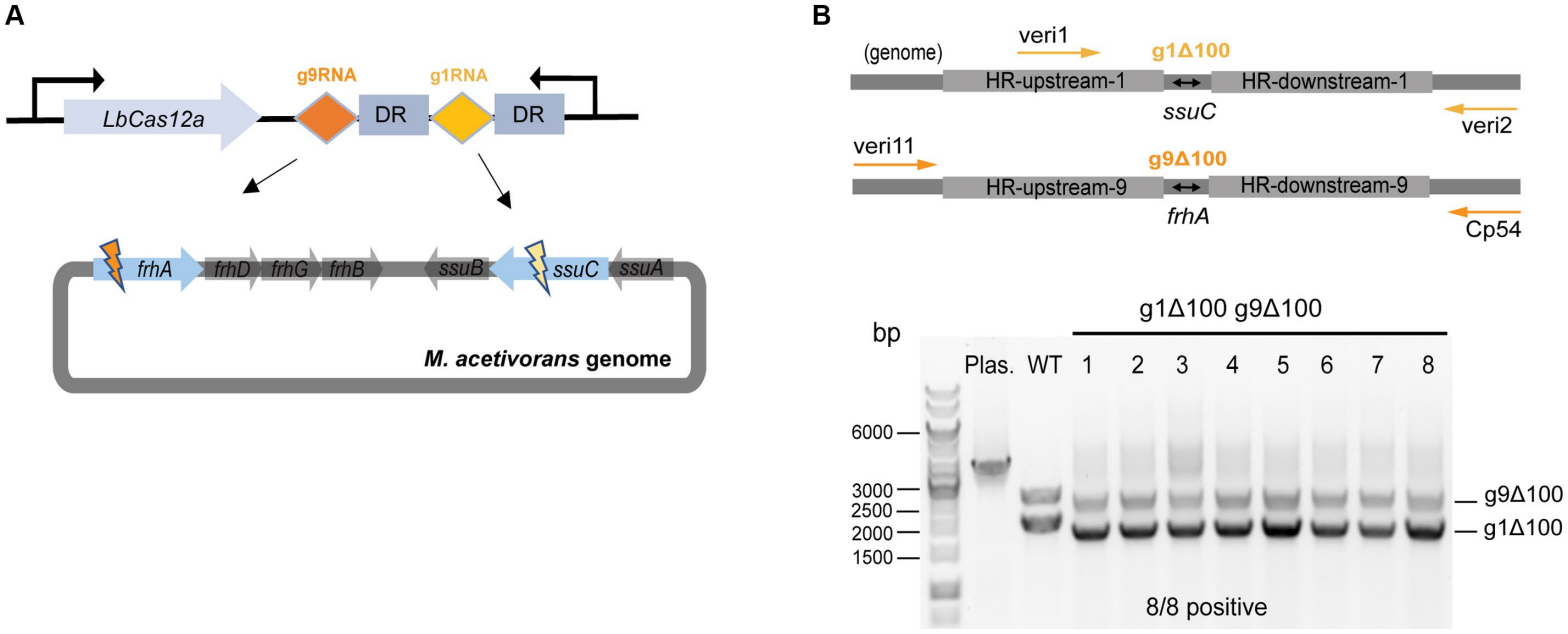
CRISPR/Cas12a-mediated multiplex genome editing. **(A)** The CRISPR array designed for targeting two sites on the genome. g1RNA and g9RNA target *ssuC* and *frhA*, generating two DSBs in the genome simultaneously. DR, direct repeat sequences in the gRNA cassette. **(B)** Editing efficiency of the multiplex gene knockout. Upper panel, scheme for the engineered genome with 100 bp-deletions in both *ssuC* and *frhA* sites and the primers used in colony PCR. veri1 and veri2, forward and reverse primer target HR sequence and the downstream of *ssuC*. veri11 and Cp54, forward and reverse primer target the upstream and the downstream of *frhA*. Bottom panel, positive rate of multiplex gene knockout. Eight transformants were randomly selected for colony PCR to verify the gene knockout efficiency. Plas. and WT are plasmid pMCp3-g1g9–100 and wild type *M. acetivorans* strain, respectively, and served as the negative controls. g1Δ100, gene deletion in *ssuC*. g9Δ100, gene deletion in *frhA*. Thermo Scientific™ GeneRuler 1 kb DNA ladder was used for sizing DNA fragments.

First, plasmid pMCp2-g1g9RNA was constructed to simultaneously target the *ssuC* and *frhA* (encoding the coenzyme F420 hydrogenase alpha subunit) genes (Figure 4A), where the gRNA cassette contains two tandem gRNA sequences (g1RNA and g9RNA) equipped with promoter P*mtaC1* and terminator T*mtaB1* (Supplementary Figure S6). Next, to assess the effect of double-site deletions, two sets of HR sequences that generate 100-bp deletions in each of the genes (see above) were designed and assembled into pMCp2-g1g9RNA, producing the plasmid pMCp3-g1g9–100. Among the obtained Pur^R^ transformants (107 ± 23 CFUs per 2 μg DNA), eight were randomly selected for colony PCR. Primers veri1/veri2 and veri11/Cp54 were used to target the genome regions flanked the *ssuC-and frhA-*breakages, separately (Figure 4B). All eight transformants were positive and the desired gene deletions were detected in both *ssuC* and *frhA* regions. Three isolates were randomly picked for Sanger sequencing and all clones were correctly edited (Supplementary Figure S7A).

To evaluate the versatility of our CRISPR/Cas12a toolbox for multiplex editing, simultaneous gene deletion and insertion at two independent sites were attempted with the plasmid pMCp3-g1-uid-g9-100. This construct facilitates the insertion of the *uidA* cassette into the *ssuC* locus as well as makes a 100-bp deletion in the *frhA* gene in one round of transformation (Supplementary Figure S7B). Out of the 280 CFUs of Pur^R^ transformants, six were randomly selected for colony PCR verification using primers veri2/veri8 and veri9/veri13 (Supplementary Figure S7C). 5/6 were edited correctly in both sites (i.e., contain the *uidA* cassette within the *ssuC* gene and exhibit a 100 bp-deletion within the *frhA* gene), which indicates the potential of using CRISPR/Cas12a system in multi-tasks genome editing in metabolic engineering applications.

### Plasmid curing

To demonstrate iterative editing with the CRISPR/Cas12a system, the plasmid curing efficiency was calculated for the positively engineered M73-uidA isolate. For plasmid removal, the isolate was cultivated in HS-methanol medium without puromycin for five serial transfers (i.e., 1% inoculum of cells in early stationary phase was utilized in each transfer). Subsequently, cells were streaked out on HS solid medium containing 20 μg/mL 8ADP for counterselection. Seven 8ADP^R^ transformants were randomly selected to detect the curing efficiency and the consistency of gene editing. As a result, 7/7 of the isolates exhibited outright plasmid removal, while 5/7 had correctly inserted *uidA* cassettes (Supplementary Figure S8). To explore a faster procedure, plasmids removal efficiency after only one round of transfer (same conditions as above) was evaluated. Same plasmid curing efficiency was obtained with the randomly selected 8ADP^R^ transformants, demonstrating the reliable application of the CRISPR/Cas12a system for performing multiple rounds of gene editing in *M. acetivorans*.

## Discussion

We developed a CRISPR/Cas12a toolbox to facilitate the genetic engineering of *M. acetivorans*. No significant effects on cell growth were observed with LbCas12a protein expression in *M. acetivorans*, allowing CRISPR/Cas12a to be a useful tool in this methanogen. Achieving a higher targeting efficiency usually requires the simultaneous screening of multiple gRNA sequences, which often involves using laborious and multi-step methods to construct several plasmids. As a simplification, we constructed *AarI*-free plasmid pMCp2-gX (Supplementary Figure S9), thereby allowing for quick preparation of multiple Cas12a-gRNA-expressing plasmids through the Gibson assembly method.

Many factors can affect the editing performance of CRISPR/Cas systems, such as the genome regions to be edited, gRNA targeting efficiency and precision, and HR sequence length. For the present study, a 23-nt gRNA was empirically chosen, as the longer sequence length displayed improved targeting specificity compared to a shorter 20-nt gRNA (Bin Moon et al., 2018; Gao et al., 2018). Conversely, shorter gRNA sequence lengths in the Cas9-mediated system proved to be more effective (Fu et al., 2014). It is worth noting that transformation efficiencies varied when using polyethylene glycol (PEG) 4,000-mediated transformation, although there was no significant effect on the positive rates.

Long fragment deletions are reported to be more efficient with the CRISPR/Cas12a system than the Cas9-based one (Wang et al., 2022), which is consistent with our experiments where the Cas12a-mediated long fragment deletion of 2000 bp resulted in a higher number of transformants than obtained with CRISPR/Cas9 editing (Nayak and Metcalf, 2017). To establish the possible length limits of gene deletion with our CRISPR/Cas12a system, the plasmid pMCp3-g9-3500 was constructed for removing the entire *frhADGB* operon, resulting in a 3,500 bp gene knockout from the genome. A higher number of Pur^R^ transformants (2,800 ± 1,400 CFUs) was obtained in comparison to the 2000 bp-deletion trial. Colony PCR and Sanger sequencing proved that all the transformants selected were edited correctly and no heterozygous genotypes were observed (Supplementary Figure S10), which suggests that our CRISPR/Cas12a editing tool is efficient for making long fragment deletions.

To assess the accuracy and effectiveness of our toolbox, five randomly selected engineered strains as well as a parent strain were sent for whole genome sequencing (WGS). All genomes contained the desired mutations. It must be mentioned, however, that intrachromosomal translocations occurred in 3/5 of the tested strains (Supplementary Table S3), while no structural variants were detected in the parent strain. These intrachromosomal translocations have not been observed in previous mutational techniques used in *M. acetivorans*. Intrachromosomal translocations arise due to the mistakes made by the repair mechanism of the cells (Brunet and Jasin, 2018). As non-homologous end joining (NHEJ) is absent in *M. acetivorans*, the possible reasons for genomic aberrations are either alternative NHEJ as known as microhomology-mediated end joining (MMEJ), or homologous recombination pathways (Agarwal et al., 2006; Nayak and Metcalf, 2017). The possible presence of MMEJ in *Methanosarcina* has been hypothesized given the presence of PARP-like enzyme (Raychaudhuri et al., 2000). Additionally, *M. acetivorans* genome contains annotated flap endonuclease (FEN) which is also involved in MMEJ pathway. Detailed understanding of MMEJ in archaea would require additional studies (Marshall and Santangelo, 2020), but MMEJ being one of the leading causes of translocation has been shown in mouse embryonic stem cells (Brunet and Jasin, 2018). The tendency of all CRISPR systems (Cas9, Cas12a, and Cas12f) to induce intrachromosomal translocations was also revealed in the editing of eukaryotic cells (Xin et al., 2022). Therefore, CRISPR tools-triggered genomic structural variations should be considered when using them for genome editing in *M. acetivorans* and as such we would recommend users for WGS after using the toolbox. Genome translocations have been important factors for the evolution and adaption of *Methanosarcinales*, where many organisms were reported with rich transposable elements and transposases (Gehlert et al., 2023). Genome annotations of *M. acetivorans* have been shown to carry more than 70 transposases (Galagan et al., 2002), indicating its potentially active transposon activity. If not the DNA repair mechanisms, another possible cause for intra-chromosomal translocations could be the transposases. By coupling it with WGS, this toolbox provides an additional tool in the repertoire for editing *M. acetivorans*.

## Materials and methods

### Strains and media

High-salt (HS) medium containing 125 mM methanol was used for cultivating *M. acetivorans* in single-cell form at 37°C (Sowers et al., 1993). *M. acetivorans* WWM73 was used as the host strain in this study and relevant derivatives are listed in Table 2. HS solid medium containing 1.4% agar (Bacto™ Dehydrated, Fisher Scientific) and 2 μg/mL puromycin (InvivoGen, Inc.) was used for screening *M. acetivorans* transformants. HS solid medium containing 1.4% agar and 20 μg/mL 8ADP (Sigma-Aldrich) was used for screening *M. acetivorans* transformants in the plasmid curing experiment. *E. coli* DH10B (Fisher Scientific) and *E. coli* XL10-Gold (Agilent Technologies) were used as the cloning hosts for constructing CRISPR plasmids. *E. coli* transformants were selected using lysogeny broth (LB) solid medium supplemented with 100 μg/mL ampicillin.

**Table 2:** Strains used in this study.

Strain	Properties	Source
*Methanosarcina acetivorans*		
WWM73	Δ*hpt*::P*mcrB*-*tetR*-φC31-*int*-attP	Guss et al. (2008)
M73-100	Δ*hpt*::P*mcrB*-*tetR*-φC31-*int*-attP, Δ*ssuC* (72928–73,101)::GCGGCCGC	This study
M73-500	Δ*hpt*::P*mcrB*-*tetR*-φC31-*int*-attP, Δ*ssuC* (72782–73,301)::GCGGCCGC	This study
M73-1000	Δ*hpt*::P*mcrB*-*tetR*-φC31-*int*-attP, Δ*ssuB* Δ*ssuC* (72532–73,551)::GCGGCCGC	This study
M73-2000	Δ*hpt*::P*mcrB*-*tetR*-φC31-*int*-attP, Δ*ssuB* Δ*ssuC* Δ*ssuA* (72032–74,051)::GCGGCCGC	This study
M73-3500	Δ*hpt*::P*mcrB*-*tetR*-φC31-*int*-attP, Δ*frhADGB* (1165689–1,169,233)::GCGGCCGC	This study
M73-100-100	Δ*hpt*::P*mcrB*-*tetR*-φC31-*int*-attP, Δ*ssuC* (72928–73,101)::GCGGCCGC, Δ*frhA* (1166033–1,166,145)::GCGGCCGC	This study
M73-uid	Δ*hpt*::P*mcrB*-*tetR*-φC31-*int*-attP, Δ*ssuC* (72928–73,101)::P*mcr*-*uidA*-T*mcr*	This study
M73-uid-100	Δ*hpt*::P*mcrB*-*tetR*-φC31-*int*-attP, Δ*ssuC* (72928–73,101)::P*mcr*-*uidA*-T*mcr*, Δ*frhA* (1166033–1,166,145)::GCGGCCGC	This study
*Escherichia coli*		
DH10B	F– *mcrA* Δ(*mrr-hsdRMS-mcrBC*) φ80*lacZ*ΔM15 Δ*lacX74 recA1 endA1 araD139* Δ(*ara-leu*)7697 *galU galK* λ– *rpsL* (*Str^R^*) *nupG*	New England Biolabs
XL10-Gold	Tet^r^ Δ(*mcrA*)*183* Δ(*mcrCB-hsdSMR-mrr*)*173 endA1 supE44 thi-1 recA1 gyrA96 relA1 lac* Hte [*F' proAB lacI^q^Z*Δ*M15* Tn*10* (Tet^r^) Amy Cam^r^]	Agilent Technologies

### Primers and plasmid construction

All plasmids used in this study are listed in Table 1. All primers are listed in Supplementary Table S1. PrimeSTAR® Max DNA Polymerase (Takara Bio) was used for amplifying gene fragments by PCR. SapphireAmp Fast PCR Master Mix (Takara Bio) was used for verifying *E. coli* and *M. acetivorans* transformants by colony PCR. HiFi DNA Assembly Master Mix (New England BioLabs) was used for Gibson assembly and T4 DNA Ligase (Promega) was used for ligation. crRNA sequences for multiplex genome targeting were synthesized and combined using the Gibson assembly method. Donor DNA with upstream and downstream of HR arms was inserted immediately downstream of the gRNA cassette using either Gibson assembly or ligation to construct the CRISPR/Cas12a genome editing system, known as plasmid pMCp3-X in Table 1.

### DNA transformation methods

Polyethylene glycol (PEG) 4,000-mediated transformation of *M. acetivorans* was performed with 2 μg DNA transformed according to the method described previously (Oelgeschläger and Rother, 2009). *M. acetivorans* transformants were grown on plates of HS solid medium with required antibiotics and incubated at 37°C for 10–15 days in an anaerobic jar with a controlled headspace of N_2_/CO_2_/1% H_2_S (75/20/5). Chemically competent *E. coli* cells were used for the transformation of assembled and ligated plasmids.

### Whole genome sequencing (WGS) of the engineered strains

The genomic DNA was extracted with Wizard® Genomic DNA Purification Kit (Promega). The Microbial WGS was performed using Illumina NovaSeq 6,000 by Novogene (UK) Company. Samples were sequenced in a paired-end 150 bp sequencing strategy and aligned with the *M. acetivorans* (C2A) reference genome (NC_003552.1) through BWA (Li and Durbin, 2009) software (parameters: mem-t 4-k 32-M). Structural variants (large deletions, insertions and translocations) were predicted using BreakDancer (Chen et al., 2009). Relevant data has been uploaded in National Center for Biotechnology Information (NCBI) with accession no. PRJNA1026875.

## Data availability statement

The original contributions presented in the study are included in the article/Supplementary materials, further inquiries can be directed to the corresponding authors.

## Author contributions

PZ, JB, and SS conceived the study. PZ and JB performed the experiments. TS and PZ analyzed the whole genome sequencing results. PZ wrote the manuscript. JB and SS supervised the experiments and revised the manuscript. All authors contributed to the article and approved the submitted version.
