# Supplementary material for: CRISPR/Cas12a toolbox for genome editing in Methanosarcina acetivorans
Source: Front Microbiol. 2023 Dec 12;14:1235616. doi: 10.3389/fmicb.2023.1235616 (PMC10750270; doi:10.3389/fmicb.2023.1235616)
Supplement: Supplementary file 1 [file Data_Sheet_1.docx]

Supplementary Material

**CRISPR/Cas12a toolbox for genome editing in**

***Methanosarcina acetivorans***

**Ping Zhu, Tejas Somvanshi,** **Jichen Bao^*^, Silvan Scheller^*^**

*** Correspondence:** Silvan Scheller, Jichen Bao

E-mail: [silvan.scheller@aalto.fi](mailto:silvan.scheller@aalto.fi), [jichen.bao@aalto.fi](mailto:jichen.bao@aalto.fi)

**
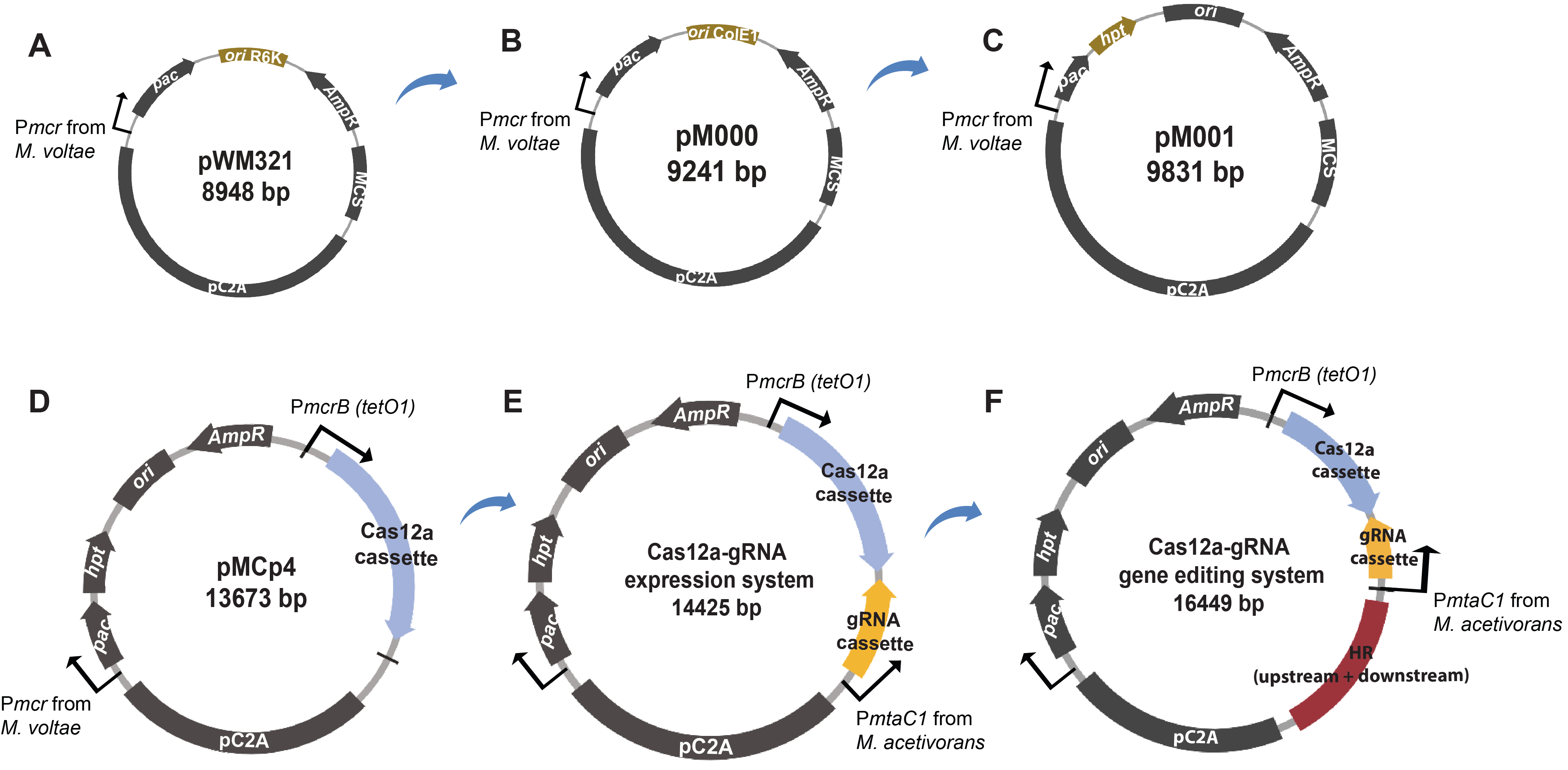
**

**Supplementary Figure 1. Plasmid maps of the Cas12a-mediated editing system.** **(A)** Plasmid pWM321, *Escherichia coli/Methanosarcina* shuttle vector. Plasmid components are shown in the map. pC2A, a naturally occurring plasmid from *Methanosarcina acetivorans*. *pac*, puromycin acetyltransferase, which is regulated by the promoter from *Methanococcus voltae* methyl reductase operon (P*mcr* from *M. voltae*). *ori* R6K, the origin of replication from plasmid R6K enables the plasmid cloning in *E. coli* strains. *Amp^R^*, β-lactamase (*bla*) gene, which confers resistance to ampicillin. MCS, multiple cloning site. **(B)** Plasmid pM000, pWM321-derived plasmid where the *ori* R6K is replaced by the *ori* from plasmid ColE1 (*ori* ColE1). **(C)** Plasmid pM001, pM001-derived plasmid where the hypoxanthine phosphoribosyltransferase (*hpt*) gene was inserted downstream of *pac* and driven by P*mcr* from *M. voltae*. **(D)** Plasmid pMCp4 expressing the Cas12a in *M. acetivorans*, where the MCS region from pM001 was replaced by the Cas12a cassette. Cas12a cassette consists of Cas12a gene from *Lachnospiraceae bacterium* (Lb) and the promoter P*mcrB* (*tetO1*). **(E)** The Cas12a-gRNA expression system generates double-stranded breaks (DSBs) in the genome. gRNA cassette, the gRNA containing spacer and direct repeat (DR) sequences was expressed by the promoter from *M. acetivorans* methanol-specific methyltransferase (P*mtaC1*). **(F)** The Cas12a-gRNA gene editing system repairs the DSB with homologous repair (HR) arms to facilitate genome editing in *M. acetivorans.*

**
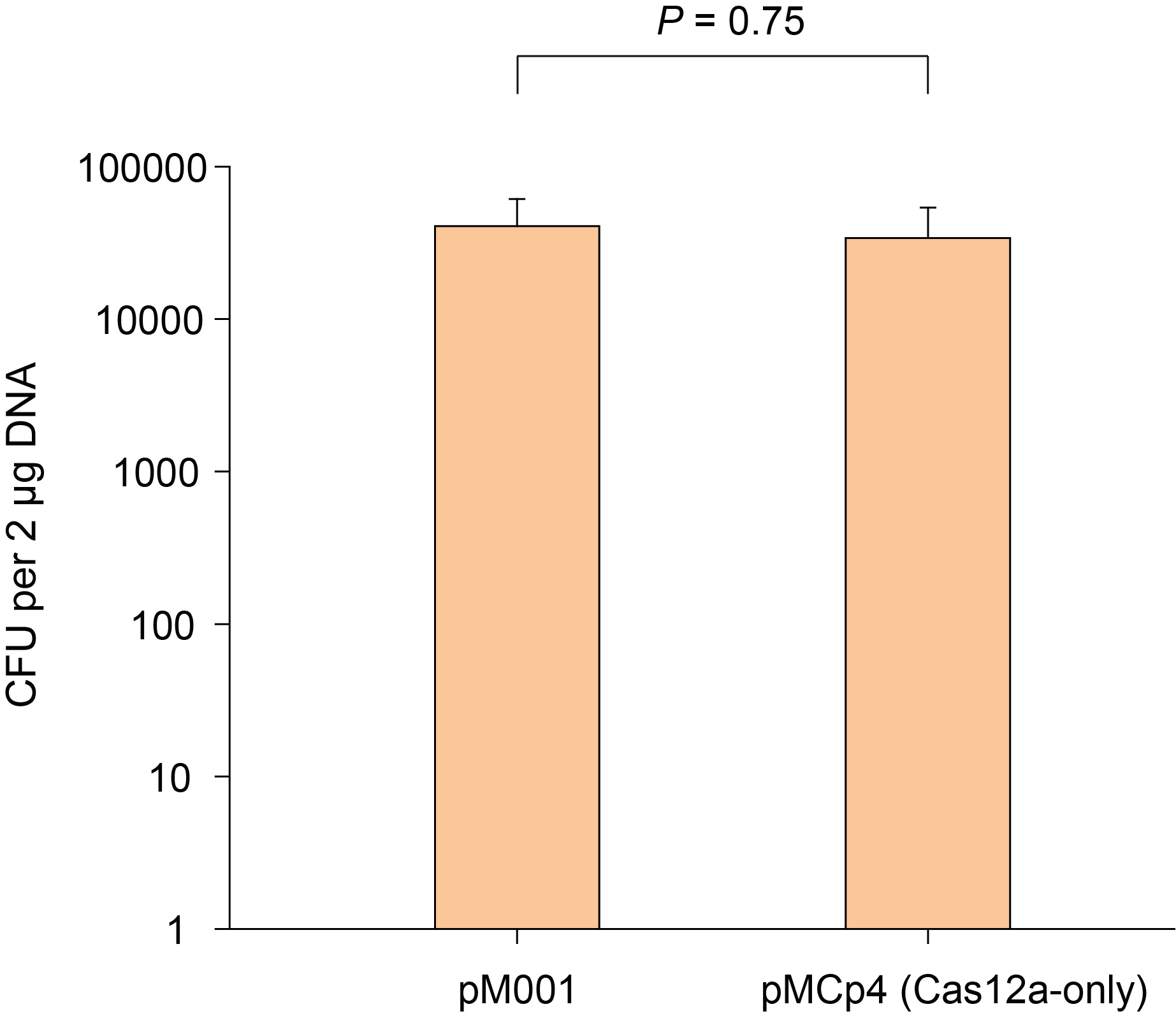
**

**Supplementary Figure 2. Assessing the toxicity of Cas12a to *M. acetivorans*.** Transformation efficiency of the empty vector pM001 and the Cas12a-expressing plasmid pMCp4. Error bar represents the standard deviation of triplicate measurements. *P* = 0.75 (two-tailed *t-test*), indicates no significant difference between the groups.


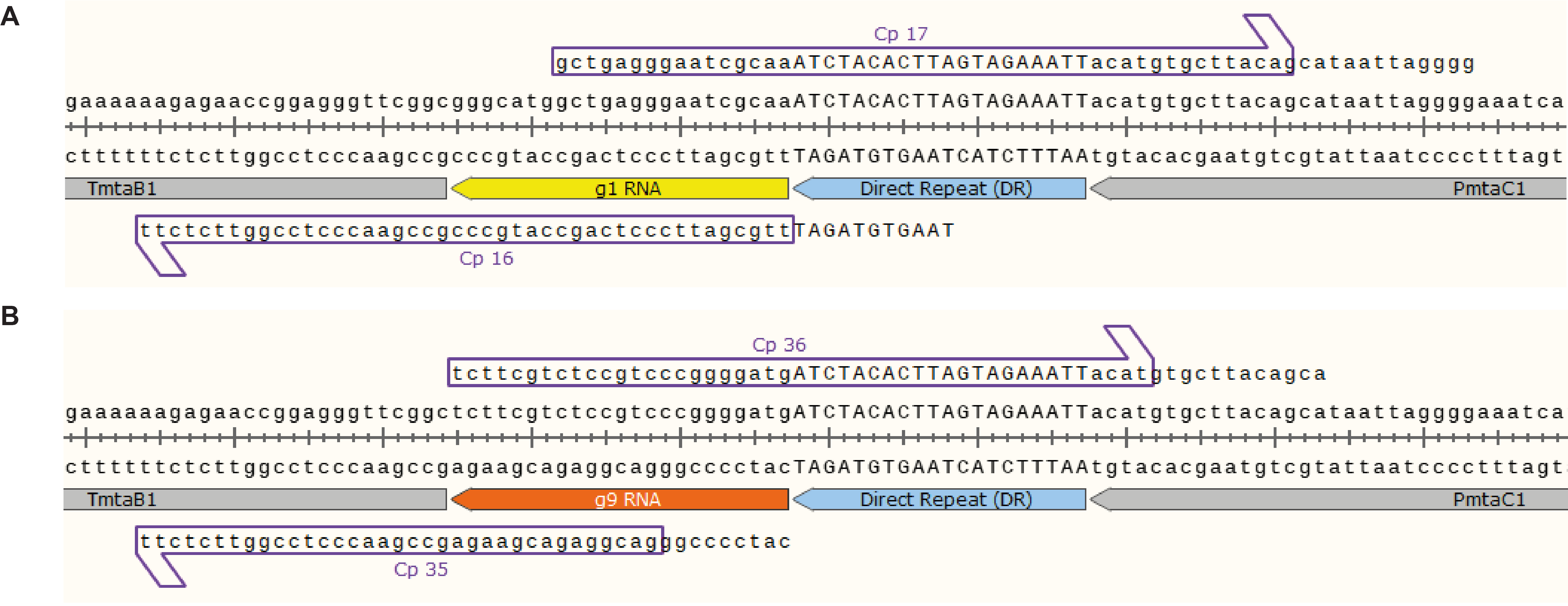


**Supplementary Figure 3. Design of gRNA cassette for Cas12a-gRNA expression system.** **(A)** The g1RNA cassette designed for targeting *ssuC*. The promoter (P*mtaC1*) and terminator (T*mtaB1*) from the methanol-specific methyltransferase operon in *M. acetivorans* and *M. barkeri* were added separately to the gRNA cassette constructs. g1RNA and DR sequences were amplified by PCR using primers Cp16 and Cp17. Cp16, forward primer for amplifying terminator T*mtaB1*. Cp17, reverse primer for amplifying promoter P*mtaC1*. **(B)** The g9RNA cassette designed for targeting *frhA*. g9RNA and DR sequences were amplified by PCR using primers Cp35 and Cp36. Cp35, forward primer for amplifying terminator T*mtaB1*. Cp36, reverse primer for amplifying promoter P*mtaC1*.


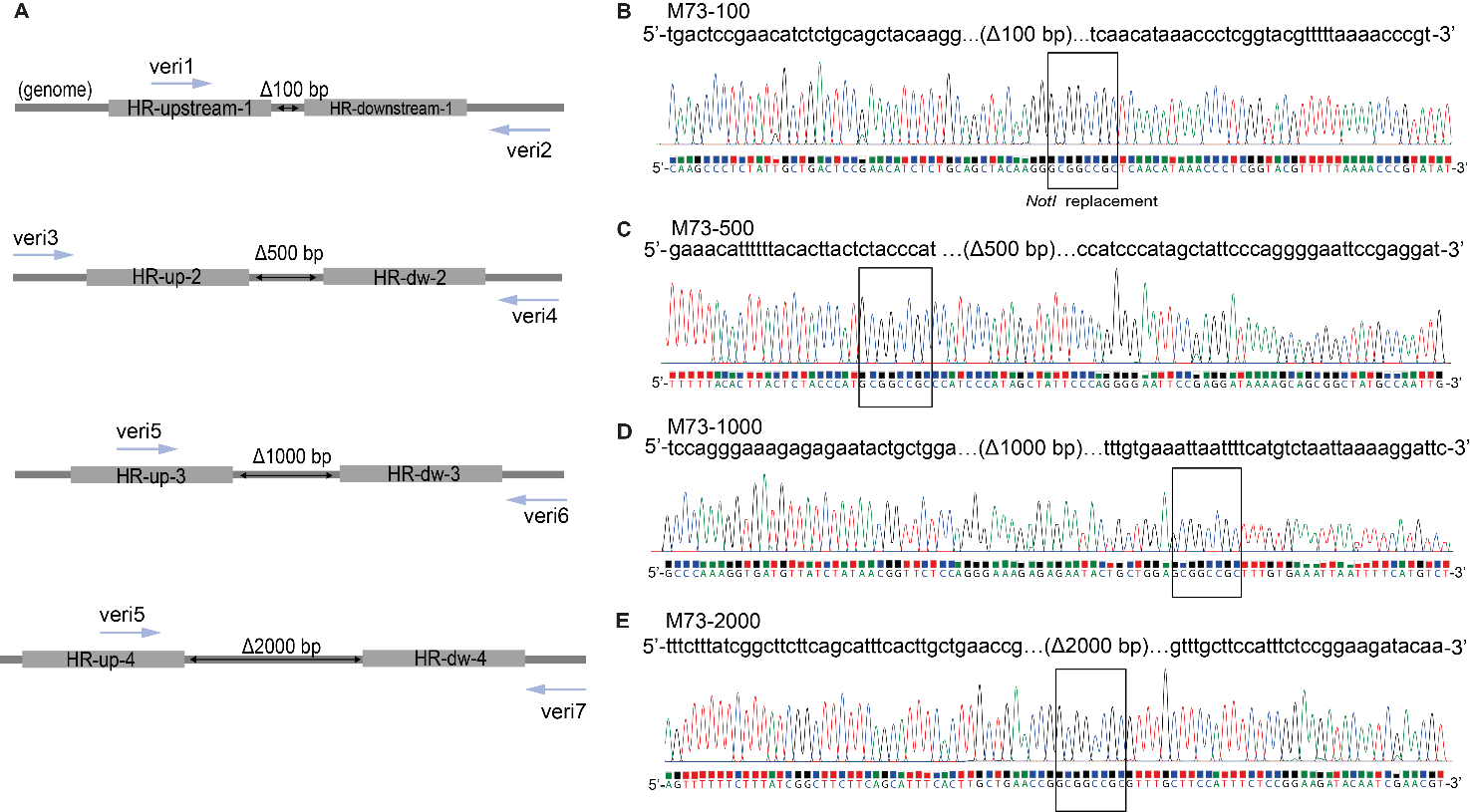


**Supplementary Figure 4. Verification of the positive clones in gene knockout experiments via colony PCR and Sanger sequencing*.*** **(A)** Genome maps of the engineered strains containing primers (blue arrow) used in colony PCR*.* The flanking HR sequences were the same as those in Figure 2B. **(B), (C), (D), (E)** Alignment of the chromatograms from Sanger sequencing results to the sequence of engineered strains to identify the deletion efficiency. The deleted region was replaced by a NotI digestion site, which is shown in black rectangle.


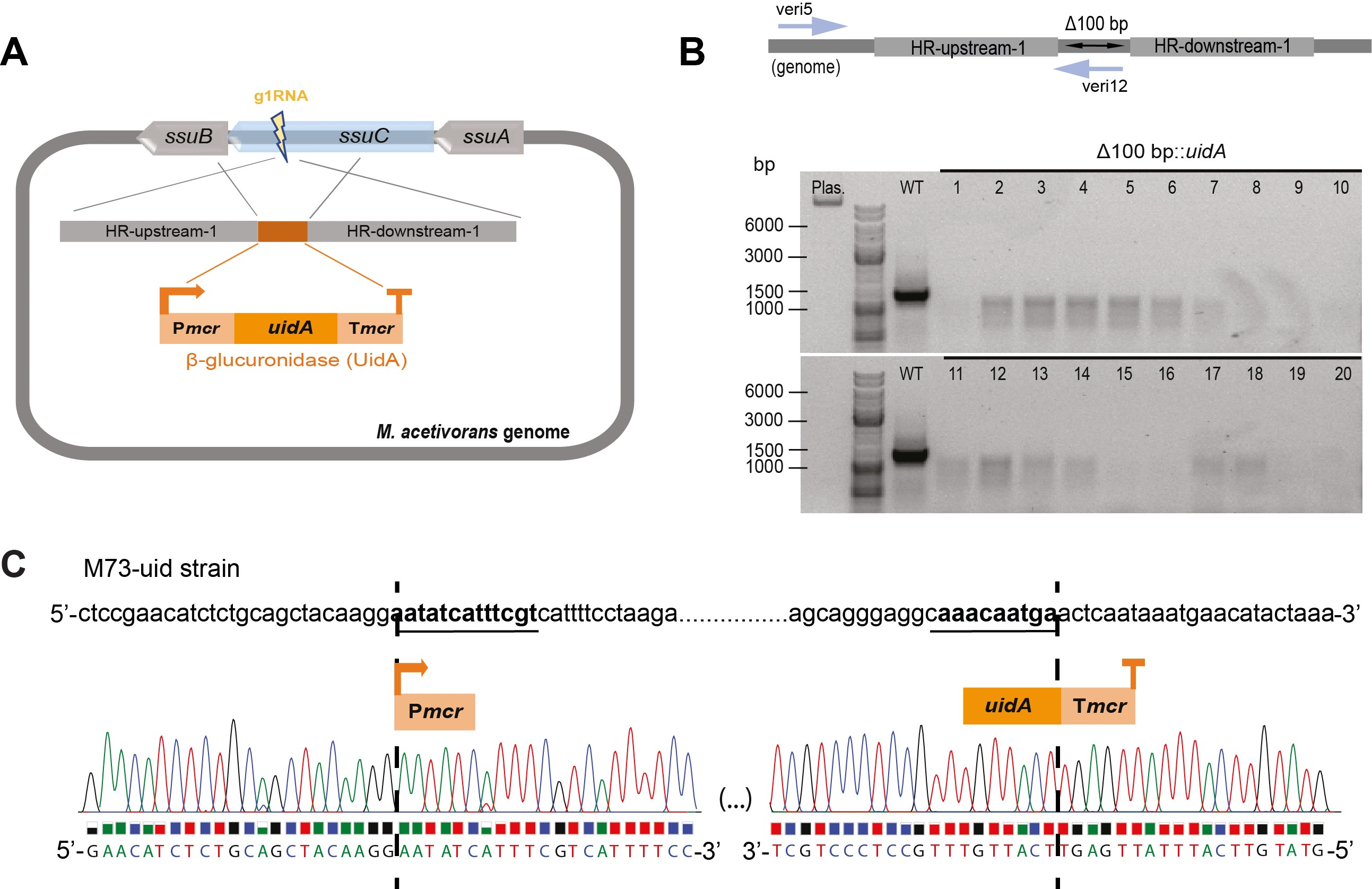


**Supplementary Figure 5. CRISPR/Cas12a-mediated** **gene insertion in *M. acetivorans.*** **(A)** Scheme for inserting *uidA* cassette into the *ssuC* locus. g1RNA used to target *ssuC* is identical to Figure 2. *uidA*, β-glucuronidase gene from *E. coli* BL21 genome. P*mcr* and T*mcr*, promoter and terminator from *M. barkeri*. **(B)** Genome map with primers (blue arrow) used in homozygous chromosome detection. veri5 and veri12, forward and reverse primers used in PCR verification targeting the upstream of the *ssuC* and the editing site, individually. Heterozygous mutant detection of the twenty randomly selected Pur^R^ transformants. Plas. and WT are plasmid pMCp3-g1-100-uid and wild type *M. acetivorans* genome served as control. Thermo Scientific™ GeneRuler DNA Ladder Mix was used for sizing DNA fragments. **(C)** Alignment of the chromatograms from the Sanger sequencing results to the sequence of mutant to identify the insertion of the *uidA* cassette. The start of the *uidA* cassette and the end of the *uidA* gene are underlined.

**
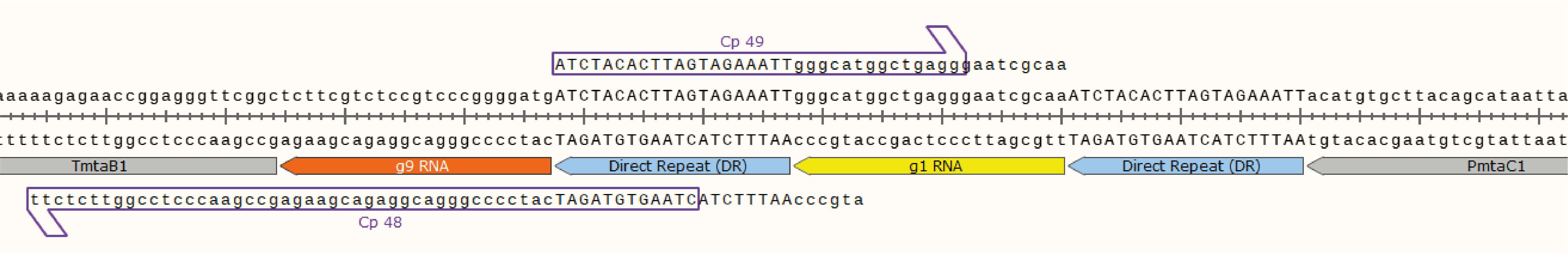
**

**Supplementary Figure 6. Design of tandem gRNAs for Cas12a-mediated multiplex genome editing.** The two gRNAs target *ssuC* and *frhA* to simultaneously generate two leakages on the genome. The promoter (P*mtaC1*) and terminator (T*mtaB1*) from the methanol-specific methyltransferase operon in *M. acetivorans* and *M. barkeri* were added to the constructs. g1RNA, g9RNA, and relevant DR sequences were amplified by PCR using primers Cp48 and Cp49 with the previously constructed pMCp2-g1RNA as the template. Cp48, forward primer for amplifying terminator T*mtaB1*. Cp49, reverse primer for amplifying promoter P*mtaC1*.


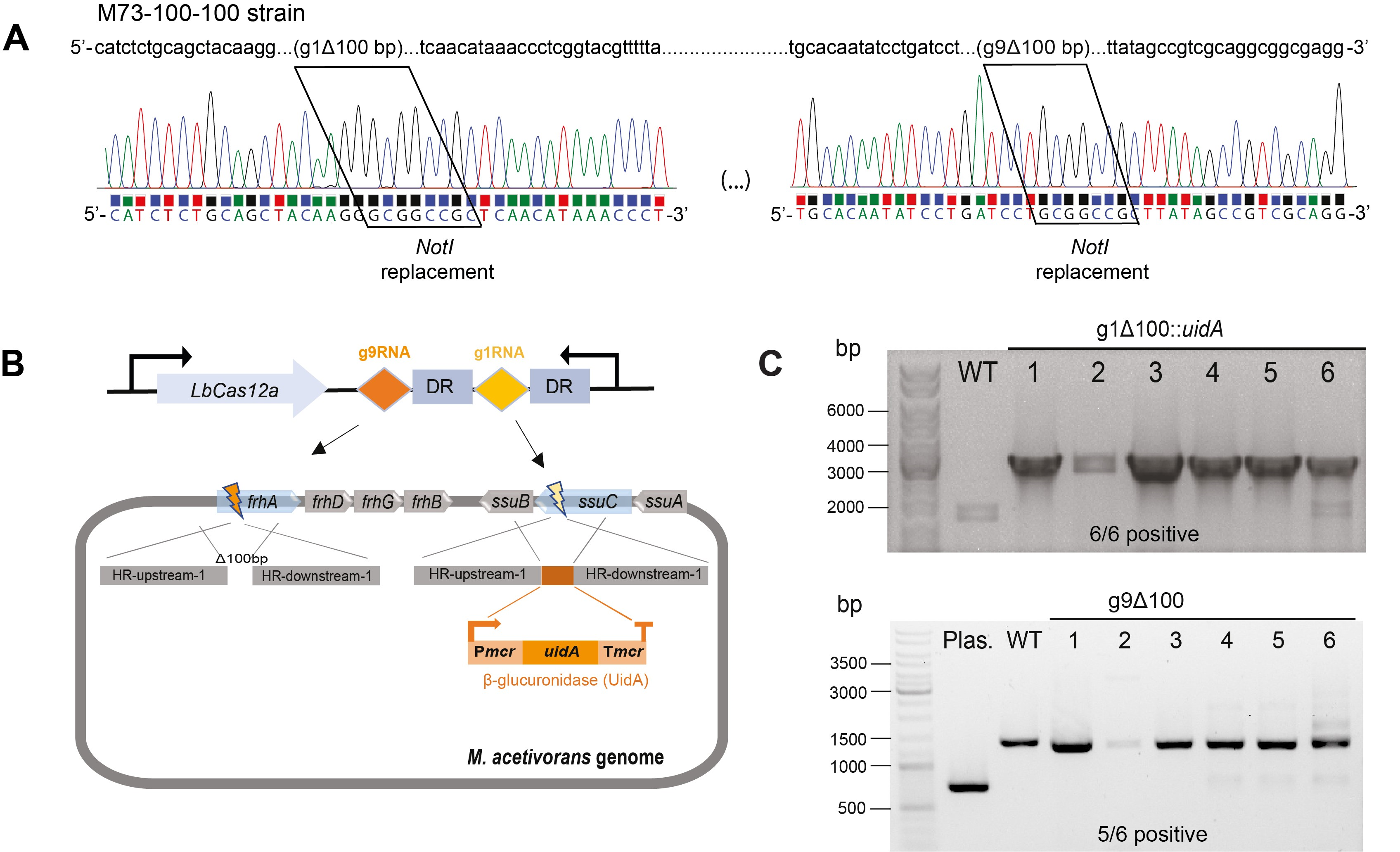


**Supplementary Figure 7. CRISPR/Cas12a-mediated multiplex genome editing in *M. acetivorans*. (A)** Alignment of the chromatograms from the Sanger sequencing results to the sequence of desired mutant to identify the simultaneous gene deletions. The deleted region is replaced with the NotI digestion site, shown in black quadrilateral. **(B)** Scheme for Cas12a-mediated simultaneous gene insertion and deletion. g1RNA-interfered targeting replaces 100 bp region with the *uidA* cassette in *ssuC* and g9RNA-interfered targeting generates 100 bp deletion in *frhA*. **(C)** Positive rate of the simultaneous gene insertion and deletion. g1Δ100::*uidA*, plasmid pMCp3-g1-uid-g9-100 generated gene insertion in *ssuC*. g9Δ100, plasmid pMCp3-g1-uid-g9-100 generated gene deletion in *frhA*. Plas. and WT are plasmid and wild-type *M. acetivorans* genome and served as negative controls. Thermo Scientific™ GeneRuler DNA Ladder Mix was used for sizing DNA fragments.


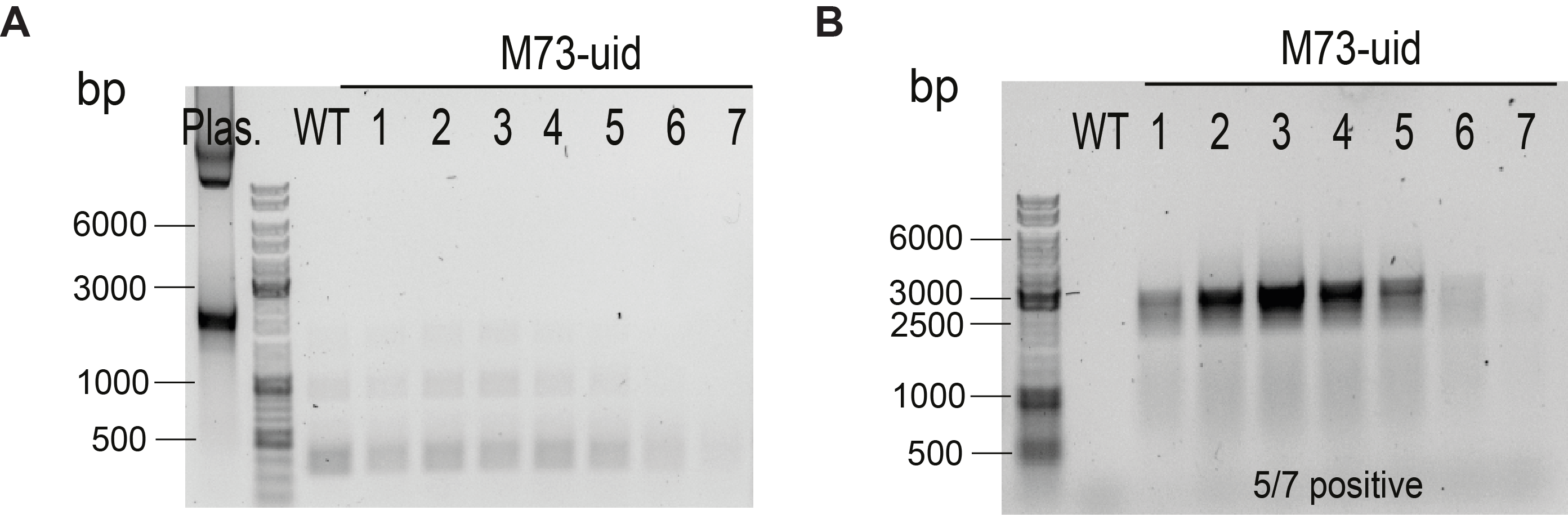


**Supplementary Figure 8. Genome editing efficiency after plasmid curing. (A)** Plasmids detection of 8ADP^R^ M73-uid isolates. Plas. and WT are plasmid pMCp3-g1-100-uid and wild-type *M. acetivorans* genome served as control in colony PCR. **(B)** *uidA* cassette detection of 8ADP^R^ M73-uid isolates. WT, wild type *M. acetivorans* genome served as control. Thermo Scientific™ GeneRuler DNA ladder mix was used for sizing DNA fragments.


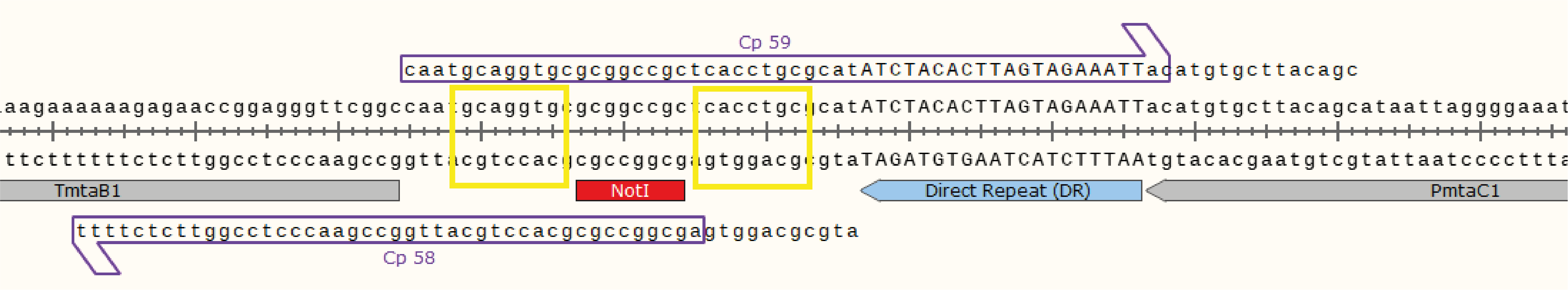


**Supplementary Figure 9. Design of exchangeable gRNA sequences for vector pMCp2-gX.** All sites for the Type IIS restriction enzyme *AarI (*an *PaqCI* isoschizomer*)* were removed from the plasmid pMCp4. The promoter (P*mtaC1*) and terminator (T*mtaB1*) from methanol-specific methyltransferase operon in *M. acetivorans* and *M. barkeri* were separately added to the gRNA cassette construct. In the gRNA cassette constructs, sites for two *AarI* and one *NotI* were placed between the DR and T*mtaB1* sequences by PCR using primers Cp58 and Cp59. *AarI* recognition sequences (5'-CACCTGC-3') are marked by yellow squares. Cp58, the forward primer for amplifying terminator T*mtaB1*. Cp59, the reverse primer for amplifying promoter P*mtaC1*.

**Protocol for constructing derived plasmids based on pMCp2-gX**

Take the construction of plasmid pMCp2-g9RNA as an example:

1. Linearize vector pMCp2-gX by digesting with *PaqCI* restriction enzyme.
2. Design oligos Cp35 and Cp36. The overlapping region between the primers is the g9RNA sequence to be assembled.
3. Prepare Cp35 and Cp36 mixture at equimolar concentration and anneal in Thermo cycler at 95°C for 5 min and slowly cool down to room temperature to amplify the overlapping DNA, of which the 5'- and 3'-ends are complementary to T*mtaB1* and P*mtaC1*, separately.
4. Gibson Assembly method is used for assembling the linearized plasmid pMCp2-gX from step 1 and the overlapping DNA product from step 3. The pMCp2-g9RNA construct is supposed to be assembled in this step.
5. Transform the assembled construct into competent *E. coli* cells and cultivate on LB agar with proper antibiotics overnight at 37°C. Screen for positive constructs by colony PCR.


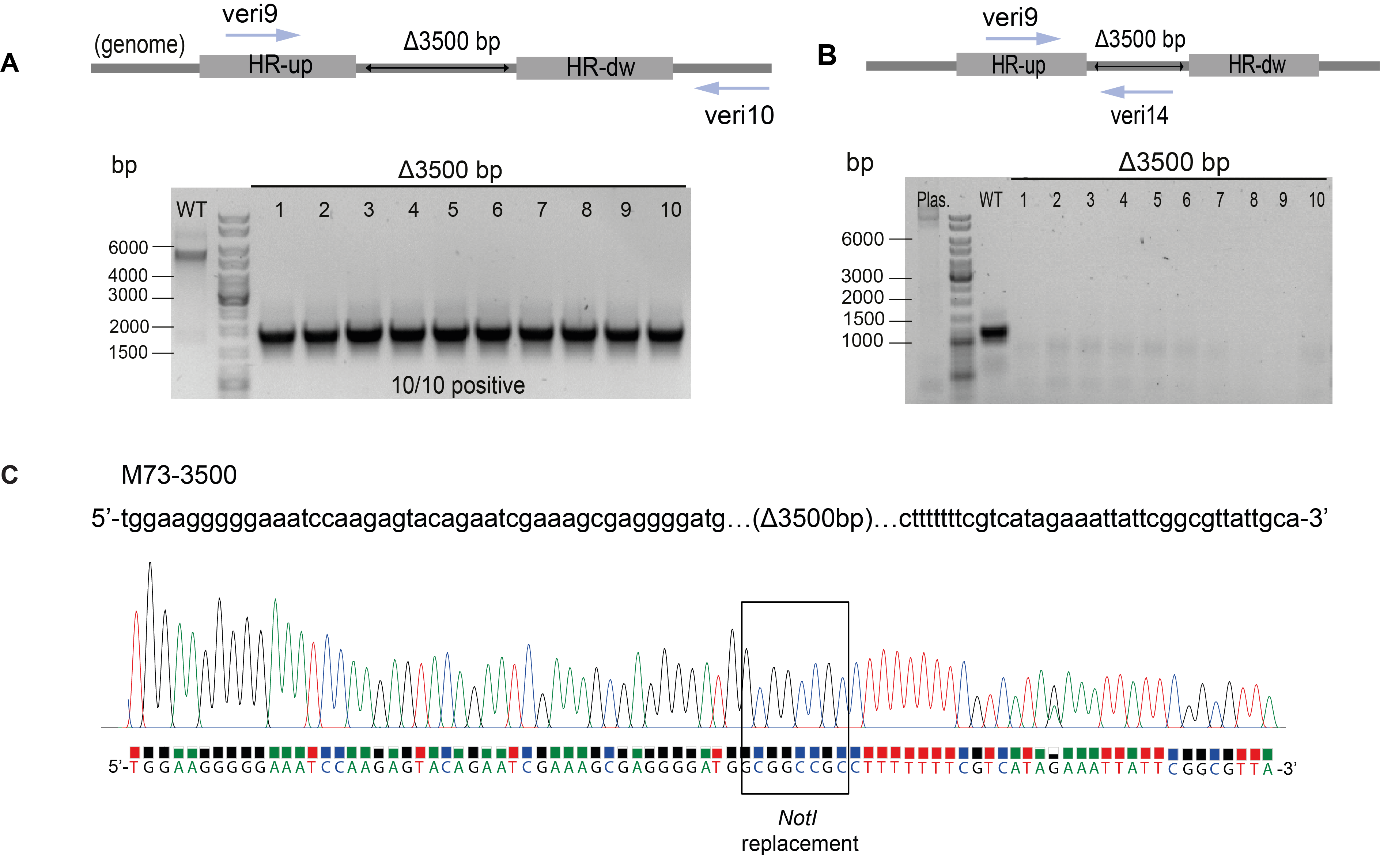


**Supplementary Figure 10. Cas12a-mediated *frhADGB* operon deletion in *M. acetivorans*.** **(A)** Editing efficiency of 3500 bp deletion-generating plasmids. veri9 and veri10 (blue arrow), forward and reward primer used in colony PCR. Ten Pur^R^ transformants were randomly selected for PCR verification. **(B)** Heterozygous detection of the ten Pur^R^ transformants with primers veri9 and veri14. Plas. and WT, plasmid pMCp3-g9-3500 and wild-type *M. acetivorans* genome served as control. Thermo Scientific™ GeneRuler DNA ladder mix was used for sizing DNA fragments. **(C)** Alignment of the chromatograms from the Sanger sequencing results to the sequence of desired mutant to identify the deletion efficiency. The deleted *frhADGB* operon was replaced with NotI digestion site, which was shown in the black rectangle.

**Supplementary Table 1. Primers used in this study**

| **Primer** | **Sequence (5’->3’)** | **Source** | **Description** |
| --- | --- | --- | --- |
| Cp1 | ctgaacacgcggcgcagttc | This study | 001-F1-F |
| Cp2 | cttccggctggctggtttattg | This study | 001-F1-R |
| Cp3 | ccggctccagatttatcagc | This study | 001-F2-F |
| Cp4 | taatggagagttgaagtgggaaggac | This study | 001-F2-R |
| Cp5 | catcgttttcctgtccttcccac | This study | 001-F3-F |
| Cp6 | catggggtcgtgcgctcctttc | This study | 001-F3-R |
| Cp7 | gaaaggagcgcacgaccccatg | This study | 001-F4-F |
| Cp8 | gaactgcgccgcgtgttcag | This study | 001-F4-R |
| Cp9 | cggcccacgtggccactagtacttctcgaggcatgcttcatttatcggagaacacaaaag | This study | Pmcr (tetO1)-F |
| Cp10 | atgaatttcctccttaatttattaaaatcattttgggactggtcacctac | This study | Pmcr (tetO1)-R |
| Cp11 | cccaaaatgattttaataaattaaggaggaaattcatatgagcaagctggagaagtttac | This study | LbCas12a-F |
| Cp12 | ggcctactctgttttaaactgttgaatttattgagtttagctggtctgggcgtactc | This study | LbCas12a-R |
| Cp13 | actcaataaattcaacagtttaaaacagagtaggcc | This study | Tmcr-F |
| Cp14 | aacccgggccctatatatggatccaatagaattatatgagcctgtaacggggat | This study | Tmcr-R |
| Cp15 | ccccgttacaggctcatataattctattcctgcagggccttttaaaaagggattgagcgaaaa | This study | T*mtaB1*-R |
| Cp16 | taagtgtagatttgcgattccctcagccatgcccgccgaaccctccggttctctt | This study | Tmta-g1-F |
| Cp17 | gctgagggaatcgcaaatctacacttagtagaaattacatgtgcttacagcataattagggg | This study | Pmta-g1-R |
| Cp18 | aattataacccgggccctatatatggatcccctgcaggaacaacatcagtcacctaaaaagagaaaac | This study | P*mtaC1*-F |
| Cp19 | gactgatgttgttcctgcaggggatcccgccggcgttagcagttttttctttatcggcttcttca | This study | HR-100up-F |
| Cp20 | aacgtaccgagggtttatgttgagcggccgcccttgtagctgcagagatgttcgg | This study | HR-100up-R |
| Cp21 | tacaagggcggccgctcaacataaaccctcggtacgtt | This study | HR-100dw-F |
| Cp22 | aggtaattataacccgggccctatatatcgccggcggtgaaggcaatggacgttcga | This study | HR-100dw-R |
| Cp23 | ggtgactgatgttgttcctgcaggggatcccgccggcgggatatccgtgtaagacccggatg | This study | HR-500up-F |
| Cp24 | cccctgggaatagctatgggatgggcggccgcatgggtagagtaagtgtaaaaaatgtttctc | This study | HR-500up-R |
| Cp25 | ctctacccatgcggccgcccatcccatagctattcccagggg | This study | HR-500dw-F |
| Cp26 | tgaggtaattataacccgggccctatatatcgccggcggaaagggctcaaaattgccactt | This study | HR-500dw-R |
| Cp27 | gtgactgatgttgttcctgcaggggatcccgccggcgaaaaaggagcttttctcgaagaagacc | This study | HR-1000up-F |
| Cp28 | ctaagaatccttttaattagacatgaaaattaatttcacaaagcggccgctccagcagtattctctctttccctgga | This study | HR-1000up-R |
| Cp29 | actgctggagcggccgctttgtgaaattaattttcatgtctaattaaaaggattcttag | This study | HR-1000dw-F |
| Cp30 | tgaggtaattataacccgggccctatatatcgccggcgagaaaggatggtggcaggaag | This study | HR-1000dw-R |
| Cp31 | gtgactgatgttgttcctgcaggggatcccgccggcgcatggaagataatctggccttctttgc | This study | HR-2000up-F |
| Cp32 | tcttccggagaaatggaagcaaacgcggccgccggttcagcaagtgaaatgct | This study | HR-2000up-R |
| Cp33 | tgaaccggcggccgcgtttgcttccatttctccggaaga | This study | HR-2000dw-F |
| Cp34 | tgaggtaattataacccgggccctatatatcgccggcgagatgtattaatacatttgggtcataatagaaaagcat | This study | HR-2000dw-R |
| Cp35 | catccccgggacggagacgaagagccgaaccctccggttctctt | This study | Tmta-g9-F |
| Cp36 | tcttcgtctccgtcccggggatgatctacacttagtagaaattacatgtgcttacagca | This study | Pmta-g9-R |
| Cp37 | ggtgactgatgttgttcctgcaggggatcctcaacaaacggtgtttcagcactgg | This study | HR-3500up-F |
| Cp38 | gaaaaaaaggcggccgccatcccctcgctttcgattctgtac | This study | HR-3500up-R |
| Cp39 | ggatggcggccgcctttttttcgtcatagaaattattcggcgttattgc | This study | HR-3500dw-F |
| Cp40 | aattataacccgggccctatatatatagttgcttggaatcttgtagttggtatgt | This study | HR-3500dw-R |
| Cp41 | gaacatctctgcagctacaaggaatatcatttcgtcattttcctaagaaa | This study | uidA cas-Pmcr-F |
| Cp42 | ggggtttctacaggacgtaacattttaatttcctccttaatttattaaaatcattttgggact | This study | uidA cas-Pmcr-R |
| Cp43 | agtcccaaaatgattttaataaattaaggaggaaattaaaatgttacgtcctgtagaaacccca | This study | uidA cas-uidA-F |
| Cp44 | ggcctactctgttttagtatgttcatttattgagttcattgtttgcctccctgctg | This study | uidA cas-uidA-R |
| Cp45 | cagcagggaggcaaacaatgaactcaataaatgaacatactaaaacagagtaggcc | This study | uidA cas-Tmcr-F |
| Cp46 | ggttttaaaaacgtaccgagggtttatgttgagggagaattatatgagcttataacggtagaaatattgttt | This study | uidA cas-Tmcr-R |
| Cp47 | tcaacataaaccctcggtacgtt | This study | uidA cas-HR-100dw-F |
| Cp48 | atgcccaatttctactaagtgtagatcatccccgggacggagacgaagagccgaaccctccggttctctt | This study | Tmta-g1g9-F |
| Cp49 | atctacacttagtagaaattgggcatggctgagggaatcgcaa | This study | Pmta-g1g9-R |
| Cp51 | tgttgttcctgcaggggatccgcactcactttggcttctgggttgcc | This study | HR-g9-100up-F |
| Cp52 | ctataagcggccgcaggatcaggatattgtgcagggcg | This study | HR-g9-100up-R |
| Cp53 | ctgatcctgcggccgcttatagccgtcgcaggcggcgag | This study | HR-g9-100dw-F |
| Cp54 | aattataacccgggccctatatatgcgggtccgaacccatcgtccgc | This study | HR-g9-100dw-R |
| Cp55 | gtgaaggcaatggacgttcgattgtatc | This study | g1HR-100dw-R |
| Cp56 | atcgaacgtccattgccttcacgcactcactttggcttctgggttgc | This study | g9HR-100up-F |
| Cp57 | gtaattataacccgggccctatatatcgccggcggcgggtccgaacccatcgtccgc | This study | g9HR-100dw-R |
| Cp58 | atgcgcaggtgagcggccgcgcacctgcattggccgaaccctccggttctctttt | This study | Tmta-gX-F |
| Cp59 | caatgcaggtgcgcggccgctcacctgcgcatatctacacttagtagaaattacatgtgcttacagc | This study | Pmta-gX-R |
|  |  |  |  |
| **Colony PCR primer** | **Sequence** | **Source** | **Description** |
| veri1 | ccacaagctccaggtatttttcca | This study | Δ100-F |
| veri2 | gaaagggctcaaaattgccactttccc | This study | Δ100-R |
| veri3 | aaaaaggagcttttctcgaagaagacctt | This study | Δ500-F |
| veri4 | agaaaggatggtggcaggaagatcttg | This study | Δ500-R |
| veri5 | ggatatccgtgtaagacccggatgcac | This study | Δ1000/Δ2000-F |
| veri6 | cagattgcatacatgactgcctatgag | This study | Δ1000-R |
| veri7 | cccttattgtgacaagcctgccagcg | This study | Δ2000-R |
| veri8 | tcccgccgggaatggtgattacc | This study | veri uidA-F |
| veri9 | aaagaaggctatcctcgaattctct | This study | Δ3500-F |
| veri10 | ctcaaatcgttagaccagaaacac | This study | Δ3500-R |
| veri11 | tgccgaagagattttcagcaagaatgtgag | This study | genome frh dele-F |
| veri12 | tcagccatgccctcgattgc | This study | g1RNA-R |
| veri13 | atcctgttctcctcgtccgttacg | This study | g9-100-R |
| veri14 | cgctccttaaaggctgctccttg | This study | g9RNA-R |

Note: gRNA sequences are underlined.

**Supplementary Table 2. Sanger sequencing results of the CRISPR/Cas12a edits**

| **Strain** | **Benching links of Sanger sequencing results** |
| --- | --- |
| ***Methanosarcina acetivorans*** | |
| WWM73 *ssuABC* operon | <https://benchling.com/s/seq-doMK6kRMy860MmS65ZQc?m=slm-Rqns8TT2OTLaORNegHkt> |
| M73-100 No.1 | <https://benchling.com/s/seq-ahATIYArhU1Pv2REZzr9?m=slm-3CbjG6FzurNOhNyBNqNh> |
| M73-100 No.2 | <https://benchling.com/s/seq-3mawiP6Rq0rWveZyvkl1?m=slm-bDUlNAUlSG4bIfR4v2SV> |
| M73-100 No.3 | <https://benchling.com/s/seq-iHTF4GSsm16WzTw7p1Xn?m=slm-2IsqdLy1ezL1j5AVVxNd> |
| M73-500 No.1 | <https://benchling.com/s/seq-yIdrV6FIV6e4n6ITdgEM?m=slm-GtI2o4QhSaPMS9M2sUF8> |
| M73-500 No.2 | <https://benchling.com/s/seq-y58TnRPdALn1LhPqeVA5?m=slm-cGD2U4wI6xvuoM0LiGUY> |
| M73-500 No.3 | <https://benchling.com/s/seq-4c5NnI0cswwdjcZ04sPP?m=slm-316PYuTkShwaXqOBcQ7U> |
| M73-1000 No.1 | <https://benchling.com/s/seq-ttB3NP9gBWgHO2pk4SmE?m=slm-YqitiFIAn86Xbd4g1Dxi> |
| M73-1000 No.2 | <https://benchling.com/s/seq-UVngyGvQqSgL1uVtkF3P?m=slm-xvuFK35Zdq3Vnvd1AZMU> |
| M73-1000 No.3 | <https://benchling.com/s/seq-5SRqD4h5TQ73HCjQuRkb?m=slm-NF7pXb4rA4zcqi1NxGa9> |
| M73-2000 No.1 | <https://benchling.com/s/seq-tMVlPULge3GUqwYoSPrY?m=slm-yoo0iw52cE6kBTrrhsFw> |
| M73-2000 No.2 | <https://benchling.com/s/seq-VyqiqwYc9mOtN7YTlVzz?m=slm-5KIouJZSO8KKVamnbypY> |
| M73-2000 No.3 | <https://benchling.com/s/seq-XUkdWeXd1iJ9zjkZ72Uk?m=slm-c2IjDXRB3Ui8vyEy6D1C> |
| M73-uid No.1 | <https://benchling.com/s/seq-FeDBPxYf7tuoO1qwoxWA?m=slm-WwPcNYapMarOGOFSeIKE> |
| M73-uid No.2 | <https://benchling.com/s/seq-PxJ1YxLyV8UnLcm4hnAp?m=slm-Hmdwek46acirq68W1QOK> |
| M73-uid No.3 | <https://benchling.com/s/seq-Py5TM4fkjivTBtdfjyg6?m=slm-JbtFWIN7kTaGNvwJpyje> |
| WWM73 *frhADGB* operon | <https://benchling.com/s/seq-d6OTilOIfGWF1VbniKFR?m=slm-JlI3E6Gpa7bW8Qp4Lc4E> |
| M73-100-100 No.1 | g1Δ100 bp:  <https://benchling.com/s/seq-tMbiGkNVHSzmQ8D51EdG?m=slm-BqxLteZwm3K9lCDP1jhb>  g9Δ100 bp:  <https://benchling.com/s/seq-4tHTsJFnYKisZGOecDsf?m=slm-6abGzsxdTIVyikfsuAuS> |
| M73-100-100 No.2 | g1Δ100 bp:  <https://benchling.com/s/seq-ZJmasFqF1LmPv5ilHHvv?m=slm-VYfW5sdmKEIKIflRqiWJ>  g9Δ100 bp:  <https://benchling.com/s/seq-XhPzdKieV4qrkw4HbkVJ?m=slm-KWIVwYTpoBazMhjtRnEq> |
| M73-100-100 No.3 | g1Δ100 bp:  <https://benchling.com/s/seq-iQz0dRZGRuDVpwx6gH2Q?m=slm-M0tm2UGwJdbJTi1go7vJ>  g9Δ100 bp:  <https://benchling.com/s/seq-wZ7g77qrOlqk0IDstDLq?m=slm-i5yCbBLBks9KgpWVArnT> |
| M73-3500 No.1 | <https://benchling.com/s/seq-NZ34yc1B7UfWdCHYcaVQ?m=slm-zoTaqr19GGODtV8sZf9K> |
| M73-3500 No.2 | <https://benchling.com/s/seq-Q13VuKURhWGilos1txQd?m=slm-lNScLiq3AP12ntqGmjjA> |
| M73-3500 No.3 | <https://benchling.com/s/seq-L6mqMtlEBgvzftQfUoql?m=slm-p3Wge5AfPhf7oem9Vket> |

**Supplementary Table 3. Whole genome sequencing results of the Cas12a-edited strains**

| **Structural variants** | ***M. acetivorans* Strain** | | | | | |
| --- | --- | --- | --- | --- | --- | --- |
|  | **WWM73 (WT)** | **M73-1000** | **M73-2000** | **M73-3500** | **M73-uid** | **M73-100-100** |
| Δ1041 bp | - | ✓ | - | - | - | - |
| Δ2047 bp | - | - | ✓ | - | - | - |
| Δ3560 bp | - | - | - | ✓ | - | - |
| Δ121 bp Δ114 bp | - | - | - | - | - | ✓ |
| INS^a^ | - | - | - | - | ✓ | - |
| ITX^b^ | - | - | - | ✓  Position  2736840- 3474842 | ✓  Position  71443- 540111,  2739078- 3477168 | ✓  Position  71981-537742,  2736840- 3474915 |

1. Insertion (INS)
2. Intra-Chromosomal Translocation (ITX)
