# Supplementary material for: CRISPR/Cas12a toolbox for genome editing in Methanosarcina acetivorans
Source: Front Microbiol. 2023 Dec 12;14:1235616. doi: 10.3389/fmicb.2023.1235616 (PMC10750270; doi:10.3389/fmicb.2023.1235616)
Supplement: Supplementary file 2 [file Presentation_1.pdf]

Supplementary Material

CRISPR/Cas12a toolbox for genome editing in *Methanosarcina acetivorans*

Ping Zhu, Tejas Somvanshi, Jichen Bao*, Silvan Scheller*

*** Correspondence:** Silvan Scheller, Jichen Bao

E-mail: silvan.scheller@aalto.fi, jichen.bao@aalto.fi

Links to plasmids used in this study as shown in Table 1

1https://benchling.com/s/seq-pWEZXP3cqv3djXk1rzVf?m=slm-9wKG6B4sBnfJul6znZo0

2https://benchling.com/s/seq-rpbRXtM7Puv8aAFTFlH8?m=slm-lFtDmdHrm0xugyHdoxdh

3https://benchling.com/s/seq-5buVJCDO4OyR0GhAYfwA?m=slm-hFW20kT2UbGgUfcTpXuH

4https://benchling.com/s/seq-izADBAaEMdSYvBDHk09V?m=slm-T2xZ3bumNIIRWsvcqSrH

5https://benchling.com/s/seq-URUem2HI8RPqj62WjEak?m=slm-QF4yGpwQmxmPQYB5jfWR

6https://benchling.com/s/seq-qrPVjE4FX2DdjUgus1oT?m=slm-kJyFA0mCeVCMWt4lIyX1

7https://benchling.com/s/seq-bAupkg2qr9EwaiBPFetx?m=slm-UMprIdMQCL5CPam6mpZT

8https://benchling.com/s/seq-xjHSe8KB2RH76sMjrdce?m=slm-9S6ZDFP51y7CeY1Afxyz

9https://benchling.com/s/seq-OJKOSAiETx0xiShReob4?m=slm-xKiWz3cQD9qyqKtoZx2H

10https://benchling.com/s/seq-0I49pjXSPd5Sk8YBYXH7?m=slm-nZKMlN7JmUfiUNqXaMcG

11https://benchling.com/s/seq-KLYbYr5cpUiSSGNzaf2L?m=slm-IvGDPoOeQhfO6MGAKNKx

12https://benchling.com/s/seq-Xhwi9vu0MLLpNVJHNTCn?m=slm-aPTMzKzXSPWACPGP8HVP

13https://benchling.com/s/seq-PmrJfDUVj8At5hKeRqkw?m=slm-WfLnAsLr043FIKPDDZdf

14https://benchling.com/s/seq-BlifsS2qR5cfKsYX4G38?m=slm-GFTovOEcsuX1LZLSHoSk

15https://benchling.com/s/seq-uRfotqoa4Wl0LuMPEBYj?m=slm-C28QBr2baTFBKYt1wD7W
